# Research progress of ghrelin on cardiovascular disease

**DOI:** 10.1042/BSR20203387

**Published:** 2021-01-22

**Authors:** Ming-Jie Yuan, Wei Li, Peng Zhong

**Affiliations:** Department of Cardiology, Renmin Hospital of Wuhan University, Cardiovascular Research Institute of Wuhan University, Hubei Key Laboratory of Cardiology, Jiefang Road 238, Wuchang, 430060 Wuhan, P.R. China

**Keywords:** cardiovascular disease, ghrelin, heart failure, signaling pathway, sympathetic activity

## Abstract

Ghrelin, a 28-aminoacid peptide, was isolated from the human and rat stomach and identified in 1999 as an endogenous ligand for the growth hormone secretagogue-receptor (GHS-R). In addition to stimulating appetite and regulating energy balance, ghrelin and its receptor GHS-R1a have a direct effect on the cardiovascular system. In recent years, it has been shown that ghrelin exerts cardioprotective effects, including the modulation of sympathetic activity and hypertension, enhancement of the vascular activity and angiogenesis, inhibition of arrhythmias, reduction in heart failure and inhibition of cardiac remodeling after myocardial infarction (MI). The cardiovascular protective effect of ghrelin may be associated with anti-inflammation, anti-apoptosis, inhibited sympathetic nerve activation, regulated autophagy, and endothelial dysfunction. However, the molecular mechanisms underlying the effects of ghrelin on the cardiovascular system have not been fully elucidated, and no specific therapeutic agent has been established. It is important to further explore the pharmacological potential of ghrelin pathway modulation for the treatment of cardiovascular diseases.

## Introduction of ghrelin

Ghrelin, a 28-aminoacid peptide, was isolated from the human and rat stomach and identified in 1999 as an endogenous ligand for the growth hormone secretagogue-receptor (GHS-R) [1]. Ghrelin and its functional receptor GHS-R1a and the unspliced, nonfunctional GHSR 1b existed in various human tissues including the heart and vascular [2]. In particular, ghrelin is synthesized and secreted by isolated murine and human cardiomyocytes [3]. In addition to regulating metabolism and appetite, ghrelin exerts wide spread physiological effects [4]; recent research has shown a strong relationship between ghrelin and the cardiovascular system [5–7]. Ghrelin has demonstrated cardioprotective effects, including enhancement of the endothelial and vascular function, inhibition of the sympathetic drive, reduction in blood pressure, prevention of atherosclerosis, inhibition of cardiac remodeling after myocardial infarction (MI), and improvement in cardiac function [4,8]. In this review, we will discuss the current evidence and potential mechanisms of ghrelin in cardiovascular disease.

## Ghrelin pathway in the heart

The ghrelin/GHS-R1a signaling pathway is complex. In cultured rat aortic smooth muscle cell, small interfering RNA-mediated GHSR knockdown suppressed the activation of Akt and ERK1/2 signaling pathway [9]. In addition, ghrelin protected the cardiomyocytes from ischemia/reperfusion injury and improved the cardiomyocyte survival by suppressing the excessive autophagy through reactive oxygen species (ROS) inhibition [10] and mammalian target of rapamycin (mTOR) induction [11]. Wang et al demonstrated that ghrelin ameliorates impaired angiogenesis of ischemic myocardium through GHS-R1a-mediated AMPK/ endothelial nitric oxide synthase (eNOS) signal pathway in diabetic rats [12]. Our team demonstrated that GHSR-1a overexpression significantly enhanced tube formation in human umbilical endothelial cells (HUVECs) under ischemia condition by regulating Akt and AMPK [13]. We also descripted the ghrelin signaling pathway in cardiac remodeling and autophagy regulation [14,15].

## Ghrelin modulates sympathetic activity and hypertension

Ghrelin actions are mediated by GHS-R1a that is expressed in peripheral tissues and central areas involved in the control of cardiovascular responses to stress. Ghrelin may have central and peripheral effects on sympathetic responses [16]. Tokudome group described the potential mechanisms of ghrelin-mediated regulation of the cardiac autonomic nervous system recently [4,7]. Intracerebroventricular injection of 1 nmol of ghrelin decreases arterial pressure, heart rate, and renal sympathetic nerve activity in conscious rabbits [17]. Ghrelin treatment decreased the cardiac sympathetic nerve activity and reduced the high mortality rate in rats after myocardial infarction [18] as well as in healthy humans [19–21]. In ghrelin knockout MI model, ghrelin treatment decreased plasma epinephrine and norepinephrine levels, indicating that endogenous ghrelin plays a crucial role in sympathetic inhibition [22]. Mager et al. reported that several ghrelin gene variations were associated with blood pressure (BP) levels in subjects with impaired glucose tolerance [23]. In a salt-sensitive rat model, continuous antagonism of GHS-R1a resulted in early elevations in blood pressure and increases in the autonomic nervous activity [24]. Circulating ghrelin concentrations are reported to be inversely correlated with BP [25]. Yu et al. investigated the relationship between ghrelin levels and hypertension and central obesity in 387 female adults; they found that hypertensive individuals exhibited lower levels of circulatory ghrelin, irrespective of the presence of central obesity [26]. The mechanism by which ghrelin regulates BP appears to be related to modulation of the sympathetic nervous system and direct vasodilatory.

## The effect of ghrelin on vascular activity and angiogenesis

The early research showed that human umbilical vein endothelial cells (HUVECs) express ghrelin and GHS-R1a mRNAs, and ghrelin inhibited fibroblast growth factor-2 (FGF-2) induced proliferation of HUVECs [27]. Hypoxia increased myocardial angiogenesis and cardiac VEGF level, and ghrelin inhibited these hypoxia-induced changes [28]. This is in conflict with another research, according to which, ghrelin stimulates HUVECs proliferation, migration, and angiogenesis through activation of ERK2 and PI3K/Akt signaling [29,30]. We also reported that ghrelin and GHS-R1a overexpression could induce angiogenesis in rats after MI; this process may be associated with the enhancement of VEGF and an anti-apoptosis effect. Furthermore, GHSR-1a overexpression significantly enhanced tube formation in HUVECs under ischemia condition [13,31] and was regulated by the GHSR-1a mediated AMPK/eNOS signal pathway [12]. However, systemic administration of ghrelin did not alter coronary angiogenesis in diet-induced obese mice [32]. Whether ghrelin is an anti-angiogenic factor or a pro-angiogenic factor is still controversial. We speculate that ghrelin plays a specific role in different cellular condition. In GHSR-1a gene knockout mice, the AMPK activity is notably down-regulated in endothelial cells (ECs) [33]. In human patients metabolic syndrome, ghrelin reverses endothelial dysfunction by increasing nitric oxide bioactivity [34], as well as in isolated small arteries taken from essential hypertensive patients [35]. In pulmonary hypertension, ghrelin levels have been found to be inversely associated with pulmonary arterial pressure [36]. Both in animal and human research, ghrelin could attenuate pulmonary vascular remodeling and decrease pulmonary artery pressure [37–39], partly mediated by the regulation of phosphorylation of glycogen synthase kinase 3 beta (p-GSK3 beta), and preventing endothelial cell damage and maintaining NO release [40].

## Ghrelin and arrhythmia

Intravenous injection of ghrelin elicited dose-related decrease in heart failure without a significant change in renal sympathetic nerve activity, which suggest that ghrelin has effect on the central nervous system [17]. We and others found that ghrelin significantly decreases the inducibility of ventricular tachyarrhythmias in rats after MI, accompanied by increased connexin43 [41–43]. Furthermore, ghrelin knockout mice showed more malignant arrhythmia and excessive sympathetic active after MI [44], indicating the endogenous ghrelin plays a crucial role in the regulation of electrical activity. The serum ghrelin level in the patients with atrial fibrillation was lower than that in the patients with sinus arrhythmia [45].

## The relation between ghrelin and coronary artery disease

Coronary heart disease is associated with atherosclerosis and inflammatory response. In recent years, ghrelin provides an attractive target for studies of atherosclerosis [46]. Ghrelin inhibits proinflammatory cytokine production in human endothelial cells [47,48], improves endothelial function [34], inhibits vascular smooth muscle cell proliferation [49], and ameliorates atherosclerosis by inhibiting endoplasmic reticulum stress [50]. GHS-R1a knockout mice showed decreased vessel intima-to-media ratio, as well as the smooth muscle cell involving Akt and ERK1/2 signaling [9]. Genetic variants of the ghrelin system are associated with susceptibility to MI and coronary artery disease by investigating seven single-nucleotide polymorphisms (SNPs) covering the GHSR region as well as eight SNPs across the ghrelin gene region in MI patients [51,52]. Increased pericardial active ghrelin content were found in ischemic heart disease patients, suggesting an increased ghrelin production of the chronically ischemic myocardium [53]. Furthermore, Serum ghrelin and VEGF-A levels were significantly higher in the good collateral group with severe coronary artery disease than that in the poor collateral group [54]. In isolated human internal mammary arteries (IMA), ghrelin caused a dose-dependent vasodilation of IMA rings [55] In contrast, plasma ghrelin levels seem to be unaffected in the pathogenesis of coronary slow flow [56]. These findings suggest that ghrelin may be an innovative therapeutic candidate for the prevention and treatment of atherosclerosis and coronary artery disease.

## Ghrelin improved heart failure and inhibited cardiac remodeling after MI

Both animal and clinical researches have showed that ghrelin improved left ventricular (LV) dysfunction and attenuated the development of LV remodeling [57–59]. We and others have previously reported that ghrelin inhibits post-infarct myocardial remodeling and improves cardiac function through anti-inflammation effect and inhibiting myocardial apoptosis [60,61]; we reviewed that GHS-R1a signaling pathway was involved in cardiac remodeling after myocardial infarction [14]. In addition, ghrelin enhanced survival and differentiation of human embryonic stem cell (hESC) in the infarcted heart [62]. Chronic heart failure hearts exhibit impaired ghrelin production and compensatory increase in GHS-R1a expression [63] as well as in acute myocardial infarction [64]; exercise training tended to increase ghrelin levels in heart failure patients [65]. However, GHS-R1a was decreased in diabetic cardiomyopathy and was positively correlated with sarcoplasmic reticulum Ca2+-ATPase 2a (SERCA2a) [66]. In particular, ghrelin could suppress cardiac fibrosis [67]; GHS-R1a deficiency increased Wnt/beta-catenin pathway activation in isoproterenol-induced myocardial fibrosis and induced inflammasome activation with the release of IL-18 [68], the cardioprotective effect of ghrelin against cardiac remodeling may through activating of JAK2/STAT3 signaling and inhibition of STAT1 signaling [69]. Furthermore, ghrelin attenuated cardiac hypertrophy in ghrelin knockout mice by activating the cholinergic anti-inflammatory pathway [70]. Chen recently reported that ghrelin inhibited endothelial-to-mesenchymal transition in a GHS-R1 a/AMPK/Smad7 dependent manner in a rat MI model [71], and ghrelin protects the skeletal muscle and the heart from ischemic damage by sustained autophagy and removes dysfunctional mitochondria [72]. Ghrelin regulates autophagy via a potentially novel mechanism involved in myocardial infarction [15]. The common variants in the GHS-R1a region are associated with parameters of left ventricular hypertrophy [73], a major risk factor for heart failure and sudden death.

## Conclusion

**Figure 1:**
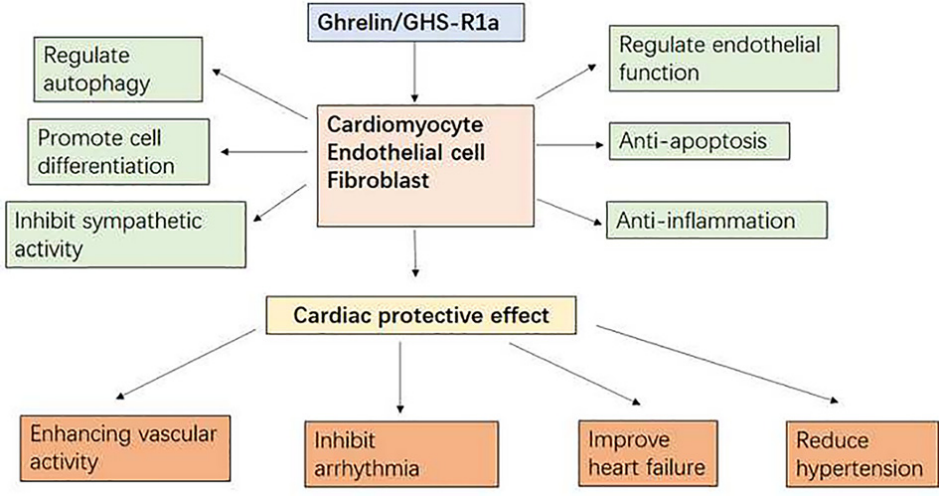
Molecular mechanisms and effect of ghrelin/GHS-R1a on cardiovascular system

In addition to stimulating appetite and regulating energy balance, ghrelin and its receptor GHS-R1a exert direct effects on the cardiovascular system, such as anti-inflammation, anti-apoptosis, inhibition of sympathetic nerve activation, regulation of autophagy, and endothelial dysfunction [4,6]. However, the molecular mechanisms underlying the effects of ghrelin on the cardiovascular system have not been fully elucidated (Figure 1), and there is no specific therapeutic agent. It is important to explore the pharmacological potential of ghrelin pathway modulation for the treatment of cardiovascular diseases.

## Abbreviations

GHS-R: growth hormone secretagogue-receptor
hESC: human embryonic stem cell
MI: myocardial infarction
mTOR: mammalian target of rapamycin
ROS: reactive oxygen species
